# Targeting carboxypeptidase A/B activity with the phosphinic inhibitor C28 reduces the asthmatic response in a mouse model of house dust mite-induced asthma

**DOI:** 10.1007/s00011-025-02046-z

**Published:** 2025-05-24

**Authors:** Venkata Sita Rama Raju Allam, David Montpeyó, Fabrice Beau, Sowsan Taha, Ida Waern, Srinivas Akula, Francesc Xavier Avilés, Julia Lorenzo, Laurent Devel, Gunnar Pejler, Sara Wernersson

**Affiliations:** 1 Department of Animal Biosciences, Swedish University of Agricultural Sciences, Uppsala, Sweden; 2 Department of Medical Biochemistry and Microbiology, Uppsala University, Uppsala, Sweden; 3 Institut de Biotecnologia i de Biomedicina (IBB) and Departament de Bioquímica i de Biologia Molecular, Universitat Autònoma de Barcelona, Bellaterra, Barcelona, Spain; 4 Centro de Investigación Biomédica en Red, Bioingeniería, Biomateriales y Nanomedicina (CIBER-BBN), 08193 Cerdanyola del Vallès, Spain; 5 CEA, INRAE, Médicaments Et Technologies Pour La Santé (MTS), SIMoS, Université Paris-Saclay, 91191 Gif-sur-Yvette, France

**Keywords:** Asthma, Inflammation, Carboxypeptidase, House dust mite

## Abstract

**Objective:**

Metallo-carboxypeptidases are implicated in several pathological contexts but their role in asthma and their potential as therapeutic targets in asthmatic settings are only partly understood. This study sought to investigate whether inhibition of carboxypeptidase activity of A and B-type could mitigate asthma-like symptoms in a mouse model of allergic airway inflammation.

**Methods:**

BALB/c mice were sensitized and challenged with repeated intranasal instillations of 10 µg house dust mite extract. Prior to each instillation, groups of mice received intraperitoneally from 0.2 to 1 mg/kg of compound 28, a phosphinic inhibitor of A/B-type carboxypeptidases. Manifestations of asthma-like features were assessed, including airway hyperresponsiveness, airway inflammation, lung histopathology and inflammatory markers.

**Results:**

Treatment with compound 28 protected against airway hyperresponsiveness and profoundly reduced the house dust mite-induced inflammation both in airways and in lung tissue. Moreover, compound 28 could mitigate airway smooth muscle and goblet cell remodelling as well as inflammatory gene expression in the lungs.

**Conclusions:**

Compound 28 could suppress multiple features of asthma in a physiologically relevant mouse model, reinforcing the potential of targeting A/B type carboxypeptidases for therapeutic purposes in allergic asthma.

**Supplementary Information:**

The online version contains supplementary material available at 10.1007/s00011-025-02046-z.

## Introduction

Asthma is a chronic airway inflammatory disorder, characterized by variable and recurring symptoms such as airflow obstruction, bronchial hyperresponsiveness, and an underlying inflammation. The pathogenesis of asthma involves a complex interplay between genetic predisposition, environmental factors and immune responses [1]. Current treatments primarily focus on preventing exacerbations and managing the symptoms through the use of inhaled corticosteroids, bronchodilators, leukotriene modifiers and biologics. Despite these therapeutic interventions, asthma remains a significant public health challenge due to its rising prevalence currently affecting around 300 million people globally and the occurrence of severe cases that are refractory to standard therapies. This underscores the need for novel therapeutic interventions that can address the heterogeneity of the disease and provide effective management for all asthma phenotypes, particularly those unresponsive to existing treatment modalities.

The M14 family of metallo-carboxypeptidases (MCPs) comprises zinc-containing enzymes that cleave off C-terminal amino acids from proteins and peptides. These MCPs are classified into subfamilies based on amino acid sequence homology and structure, of which the A/B subfamily (M14A), and the N/E subfamily (M14B) are of major importance for human physiology. The M14A subfamily includes the mast cell-restricted carboxypeptidase A3 (CPA3) [2, 3], the pancreatic CPA1, CPA2, and CPB1, the intestinal CPO, and the plasma MCP, CPB2, which is also denoted thrombin-activatable fibrinolysis inhibitor (TAFI), or CPU. They have distinct but related genomic structures, tissue locations and roles, as well as specificity profiles [2]. Another subfamily, M14B, includes CPD, CPE, CPM, CPN and CPZ that act as regulatory MCPs [4, 5]. MCPs play critical roles in various biological processes, such as the digestion of dietary proteins, controlling fibrinolysis, processing prohormones and neuropeptides, and mediating inflammatory responses [4, 5]. Based on this, MCPs are currently emerging as potential targets for therapeutic intervention in various pathological contexts [4, 5].

Overall, there is only limited knowledge on whether the M14A family of MCPs can have an impact on asthma or related lung disorders. Notably though, a series of clinical investigations have suggested that there is an association between CPA3 expression and asthma [6–8] and other chronic lung diseases [9]. Moreover, both clinical and experimental studies support a role for TAFI (CPB2) in lung inflammation, including asthma. Similarly, a clinical study revealed increased levels of TAFI in plasma of asthma patients, which was decreased following allergen exposure [10]. Increased levels of TAFI have also been observed in nasal lavage fluids from chronic rhinosinusitis (CRS) patients with asthma versus CRS patients without asthma [11]. Further, in an experimental study of LPS-induced acute lung injury, higher inflammatory responses and C5a levels were found in TAFI-deficient versus wild-type mice, suggesting a protective role of TAFI [12]. A protective role for TAFI has also been reported in an ovalbumin-based mouse model for allergic asthma [13]. It is noteworthy that TAFI in human blood is a proenzyme requiring cleavage for activation, and that its half-life there is only of 5–10 min [14]. Together, these findings suggests that MCPs of the M14A family may contribute to the manifestations of asthma and other inflammatory lung diseases, although their mechanism of action are not fully understood. Additionally, another MCP, angiotensin-converting enzyme 2 (ACE2), of acute medical protagonism in the last years through the COVID pandemics, via its presence in the respiratory track epithelium of the hosts [15], could be added to this landscape. One potential indication is that increased expression levels of ACE2 are found in sputum cells of severe asthmatics [16].

To evaluate the role of MCPs in allergic asthma, we recently assessed the impact of *Nerita versicolor* MCP proteinaceous inhibitor (NvCI), a potent and specific natural CPA inhibitor [17], in a physiologically relevant asthma model based on sensitization with house dust mite (HDM) extract. This study revealed that NvCI suppressed the AHR and goblet cell hyperplasia in sensitized animals [18]. These findings implied that CPA activity related to the M14A family of MCPs contributes to asthma pathology and, considering its association to human asthma and location in the lung mucosa, mast cell CPA3 (a CPA-like protease) was considered as a primary candidate that might fulfil such a role. However, a recent study demonstrated that genetic deletion of CPA3 in mice had no effect on key features of asthma in two experimental models [19]. These findings therefore suggest that M14A-family MCPs other than CPA3 represent primary effector MCPs in the regulation of asthma features. To advance our knowledge of the role of MCPs in asthma, and to approach a future clinical usage of MCP inhibitors in the treatment of such conditions, we here assessed the impact of two MCP inhibitors with specificity for CPA + CPB (the first) or for CPA (the second)—compound 28 (C28) [20] and compound 8 (C8) [21]—as active site-directed small-molecule drugs. They have both a pseudo peptide core and a phosphoryl function to chelate the catalytic zinc ion [20, 21] (see Supplementary Fig. 1 for exact chemical structure), and have been assayed in an experimental mouse model of asthma. We show that C28 but not C8 exhibited significant protective effects against the cardinal features of asthma, supporting the potential use of MCP inhibitors in future asthma treatment.

## Materials and methods

### Measurement of IC50 for compound 8 and 28

CPA and CPB inhibition assays were performed in 96-well plates (Corning), in presence of 1.5 nM of bovine CPA1 (Sigma) or pig CPB (Sigma) with respectively 100 µM of N-(4-Methoxyphenylazoformyl)-L-phenylalanine-OH potassium salt (AAFF, BACHEM) or N-(4-Methoxyphenylazoformyl)-L-Arginine-OH potassium salt (AAFA, BACHEM) in a final volume of 100 µl of respectively 20 mM Tris–HCl (pH 7.5), 500 mM NaCl and 0.05% w/v Brij®-35 or 20 mM Tris–HCl (pH 7.5), 100 mM NaCl and 0.05% w/v Brij®-35. Enzyme and inhibitors were incubated for 15 min before the initiation of the reaction by substrate addition. Assays were carried out at 25 °C and absorbance was read at 355 nm every 30 s for 15 min in a Multiskan Go multiwell microplate reader (Thermo Scientific). For each inhibitor, percentage inhibition was determined in duplicate experiments at different inhibitor concentrations, chosen to observe a 20–80% range of inhibition.

In the case of TAFI, inhibition assays were performed in presence of 10 nM of activated human TAFI with 100 uM of N-(4-Methoxyphenylazoformyl)-L-Arginine-OH potassium salt (AAFA, BACHEM) in a final volume of 100 ul of 20 mM Tris–HCl (pH 7.5), 100 mM NaCl and 0.05% w/v Brij®-35. Human TAFI was first activated 30’ at 25 °C at a concentration of 250 nM in presence of 20 nM of human Thrombomodulin (Sigma) and 10 nM of human Thrombin (Sigma). Activated enzyme and inhibitors were incubated for 15 min before the initiation of the reaction by substrate addition.

All Assays were carried out at 25 °C and absorbance was read at 355 nm every 30 s for 15 min in a Multiskan Go multiwell microplate reader (Thermo Scientific). For each inhibitor, percentage inhibition was determined in duplicate experiments at different inhibitor concentrations, chosen to observe a 20–80% range of inhibition.

### HDM-induced mouse model of asthma

Female BALB/c mice (8–9 weeks of age) were purchased from Taconic Biosciences (Lille Skensved, Denmark). All procedures were performed at the Swedish University of Agricultural Sciences animal facility under protocols compliant with the EU Directive 2010/63/EU for animal experiments and approved by the local ethical committee (Uppsala djurförsöksetiska nämnd; Dnr 5.8.18-12873/2019). Mice were acclimatized for one week prior to experiments. Mice were lightly anaesthetized with isoflurane using a portable isoflurane vaporizer. Mice were then intranasally challenged with 10 μg of house dust mite (HDM; *Dermatophagoides pteronyssinus*, CiteQ BV, Groningen, Netherlands) extract reconstituted in 30 μl PBS twice a week for three weeks. Control mice received 30 μl PBS intranasally. We performed two independent approaches to determine the efficacy of the carboxypeptidase inhibitors C8 and C28, described earlier [20, 21]. In the initial approach, C8 and C28 were administered intraperitoneally (0.2 mg/kg in PBS) 30 min before each PBS and/or HDM instillation. In the second approach, C28 was administered intraperitoneally (0.2 mg/kg or 1 mg/kg in PBS) 30 min before each PBS or HDM instillation. As vehicle controls, PBS was injected intraperitoneally 30 min prior to each PBS or HDM instillation.

### Measurement of airway hyperresponsiveness

Mice were anaesthetized with pentobarbital sodium (50 mg/kg) via the intraperitoneal route, 24 h after the last HDM instillation. Mice were cannulated with a 20-gauge blunt needle and were connected to a small ventilator (Buxco® FinePointe Resistance and Compliance, Winchester, UK) to record the airway hyperresponsiveness (AHR). Mice were ventilated at 160 breaths/min with a tidal volume of 0.25 mL. Lung resistance (R_L_) and dynamic compliance (Cdyn) were recorded in response to 0–50 mg/mL methacholine, as previously described [22].

### Bronchoalveolar lavage and differential cell counts

After measuring AHR, bronchoalveolar lavage (BAL) fluid was collected from the cannulated lungs by inflating the lungs twice with 0.5 mL of sterile Hanks Balanced Salt Solution (HBSS). The collected BAL fluids were centrifuged at 600×*g* for 10 min at 4 °C. Cell pellets were resuspended in 1 mL sterile HBSS for enumeration of total and differential cell counts. For total cell counts, a hemocytometer was used. For differential leukocyte counts, cytospin slides were prepared by centrifugating 100 µl cell suspensions onto glass slides at 26×*g* for 5 min using a cytospin centrifuge. Cells were stained with May Grünwald/Giemsa, and a minimum of 200 cells per slide cells were counted.

### Lung histology

Lung histology was assessed by haematoxylin and eosin (H&E) staining and airway inflammation was assessed by a tissue scoring protocol [22,23]. Eosinophils around the airways was visualized by chromotrope 2R staining [22]. Goblet cell hyperplasia, mucus production and smooth muscle cell layer thickness was visualized by periodic acid-Schiff (PAS) staining [22].

### Quantitative RT-PCR analysis

Quantitative RT-PCR (qPCR) analysis was performed as described [22]. The primers (Ccl11, Ccl8, Ccl9 and Clec7a/Dectin1) were validated by the manufacturer (BioRad, Hercules, CA). The GAPDH primers were validated in house and the sequences were as follows.

**Table:** 

GAPDH	F: TCA ACA GCA ACT CCC ACT CTT
	R: ACC CTG TTG CTG TAG CCG TAT

### In vivo and ex vivo administration of C28 for inhibitory assays in lungs and plasma

To investigate the in vivo inhibitor efficacy of C28 against MCP activity, blood and lungs were collected thirty minutes after intraperitoneal injection of naïve mice with C28 (1 mg/kg) or from untreated naïve mice as controls. Plasma was separated and stored at–80 °C. Lung lobes were immediately flash frozen using liquid nitrogen and then stored at– 80 °C. To investigate the ex vivo inhibitory effect of C28 during asthmatic and normal conditions, lungs collected from HDM-instilled and naïve mice were flash frozen and stored at– 80 °C until used for preparation of lung homogenates. Lung homogenates were treated in triplicates with 0.1, 1 or 10 nM C28, and untreated samples were used as controls in inhibition assays.

### Lung extract preparation

Lung lobes taken directly from dry ice were weighed and sheared in a Dounce homogenizer with ice-cold lysis buffer at a concentration of 50 mg of tissue per mL of buffer. The lysis buffer contained 10 mM Tris–HCl (pH 7.4), 4 M NaCl, 0.1% w/v PEG 3350 and 1X of EDTA-free protease inhibitor cocktail (Roche Diagnostics, Mannheim, Germany). Homogenates underwent vortex agitation for 15 min and were subsequently centrifuged at 16,000×*g* for 20 min. Supernatants were used immediately for activity assays or were frozen in liquid nitrogen and stored at − 80 °C for further analysis.

### Measurements of CPA inhibition

CPA activity assays were performed in 96-well plates, in presence of 0.677 nM of bovine CPA1. Lung homogenates and plasma samples were diluted to 200 µg of total protein in reaction buffer, and plated in triplicate. A fixed volume of substrate at a final concentration of 100 µM was added to initiate the reactions. Plates were incubated at 37 °C and absorbance was read at 340 nm every minute for 90 min in a Victor3 multiwell microplate reader (Perkin Elmer, Waltham, MA). The activity buffer contained 20 mM Tris–HCl (pH 7.5), 500 mM NaCl, 1% v/v DMSO and 0.05% w/v Brij®-35. The substrate used was N-(4-Methoxyphenylazoformyl)-L-phenylalanine-OH potassium salt (AAFP), and a stock solution of 1 mM was prepared in activity buffer.

### Measurements of CPB inhibition

CPB activity and inhibition assays were performed in 96-well plates, in the presence of 3 nM of CPB1 from porcine pancreas. For the activity assays, homogenates and plasma samples were diluted to 200 µg of total protein in reaction buffer, and plated in triplicate; for the inhibition assays, homogenates were also diluted to 200 µg of total protein in reaction buffer, and plated in triplicate in presence of 0.1, 1 or 10 nM of C28 (untreated conditions were used as controls). A fixed volume of substrate at a final concentration of 100 µM was added to initiate the reactions. The plates were incubated at 37 °C, and absorbance was read at 340 nm every minute for 90 min in a Spark Multimode Microplate Reader (Tecan, Switzerland). The activity buffer contained 20 mM Tris–HCl pH 7.5, 100 mM NaCl, 1% v/v DMSO and 0.05% w/v Brij®-35. The substrate used was N-(4-Methoxyphenylazoformyl)-Arginine-OH potassium salt (AAFA), and a stock solution of 1 mM was prepared in activity buffer.

### Measurements of ACE2 inhibition

ACE2 activity and inhibition assays were performed in 96-well plates, in presence of 3 nM of mouse recombinant ACE2. For the activity assays, homogenates and plasma samples were diluted to 200 µg of total protein in reaction buffer, and plated in triplicate in; for the inhibition assays, homogenates were also diluted to 200 µg of total protein in reaction buffer, and plated in triplicate in presence of 0.1, 1 or 10 nM of C28 (untreated conditions were used as controls). A fixed volume of substrate at a final concentration of 10 µM was added to initiate the reactions. The plates were incubated at 37 °C, and fluorescence was read (excitation at 320 nm and emission at 405 nm) every minute for 90 min in a Spark Multimode Microplate Reader (Tecan, Switzerland). The activity buffer contained 50 mM Tris–HCl (pH 7.5), 1M NaCl. The substrate used was Mca-YVADAPK(Dnp)-OH Fluorogenic Peptide Substrate.

### Statistical analyses

All data are represented as mean values ± SEM and were analyzed using GraphPad Prism 8.0. Two-way ANOVA analysis was performed to compare the in vivo lung function measurements between the experimental groups in response to the methacholine dose–response curve, using the Tukey post-hoc analysis. For the other experimental data, one-way ANOVA was performed to compare the difference between the treatment groups, using Tukey post-hoc multi-comparison analysis. Data was considered as statistically significant when *p*-values were ≤ 0.05.

## Results

### Inhibitory efficacy of compounds C8 and C28 against CPA and CPB activity, and of C28 against ACE2 activity

The inhibitory efficacy of the two compounds, C8 and C28, was evaluated by measuring their half-maximal inhibitory concentrations (IC50) values for inhibition of CPA1, CPB1 and CPB2/TAFI, which are displayed at Table 1, together with previous reference analysis, particularly on previous publications on the synthesis and characterization of C28 and C8 inhibitors [20, 21]. These analyses showed that C8 could potently inhibit CPA2 at nM concentrations but was unable to efficiently block the proteolytic activity of CPB1 and CPB2, confirming that C8 is highly specific for CPA activity. In contrast, C28 could efficiently inhibit CPA1, CPA2 and CPB1 and in a minor extent CPB2, demonstrating that C28 exhibits broader inhibitory activity against both CPA and CPB activity. The inhibitor constant for C28 against ACE2 activity is also displayed in Table 1, from Ref [20].

**Table 1:** Inhibitor constant (K_i_) or half-maximal inhibitory concentrations (IC50) for compounds 28 and 8 against CPA1, CPA2, CPB1, CPB2/TAFI, and ACE2^a^

Inhibitor	K_i_^b^ or IC50^c^ (nM)				
	bCPA1^b^	bCPA2^c^	pCPB1^c^	hCPB2/TAFI^c^	hACE2^b^
C28	0.5	< 0.1	0.6	98	0.15
C8	0.25	3.9	> 10,000	> 10,000	ND

^a^TAFI, thrombin-activatable fibrinolysis inhibitor; b, bovine; p, porcine; h, human; ND, not determined

^b^Values from A. Mores et al., 2008, J Med Chem and G. Covaleda et al., 2019, J Med Chem, on Refs. [20, 21]

^c^Experimental values (from this work)

### C28 but not C8 at low dose exhibits partial protective effects against HDM-induced inflammatory responses

In initial experiments, we performed a small-scale direct comparison of C8 and C28 for their ability to suppress HDM-induced airway inflammation and AHR. For this, we administered low doses of the two MCP inhibitors (C8 and C28; 0.2 mg/kg) via the intraperitoneal route (outlined in our experimental scheme; Suppl Fig. 2A). As expected, exposure of mice to HDM induced a pronounced increase in airway inflammation as demonstrated by an increase in BAL total cells, eosinophils, lymphocytes, neutrophils and macrophages (Suppl Fig. 2B–F). No signs of inflammation were observed in control groups treated with C8 and C28 alone (Suppl Fig. 2B–F). Further, treatment of HDM-challenged mice with C28 resulted in a reduction of the numbers of lymphocytes infiltrating the airways (Suppl Fig. 2D), and also a reduction of the tissue inflammation and tissue eosinophilia (Suppl Fig. 2G–H). In contrast, C8 did not cause any reduction of airway inflammatory parameters (Suppl Fig. 2B–H).

The effects of low doses of C8 and C28 on HDM-induced airway hyperresponsiveness (AHR) was also assessed. An increased lung resistance (R_L_) (Suppl Fig. 3A) with no change in dynamic compliance (Cdyn) (Suppl Fig. 4A) were observed in HDM group when compared to PBS. Furthermore, the analyses also revealed that C28, but not C8, tended to reduce the HDM-induced AHR. The effects of low doses of C8 and C28 on HDM-induced goblet cell hyperplasia was also assessed. As seen in Suppl Fig. 3B, HDM challenge led to marked goblet cell hyperplasia (as evidenced by periodic acid-Schiff (PAS) staining), but the low-dose administration of either C8 or C28 did not significantly affect the goblet cell hyperplasia. Notably though, a trend of reduced goblet cell hyperplasia was observed in response to C28 (Suppl Fig. 3B).

Together, this small-scale direct comparison of C8 and C28 indicated that C28 has a dampening effect on airway inflammatory parameters in an asthmatic setting, whereas C8 appeared to be without effect. In the next set of experiments, we therefore performed a more in-depth analysis of the effect of C28 on inflammatory parameters and AHR induced by HDM sensitization.

### C28 has profound protective effects against HDM-induced inflammation

**Fig. 1:**
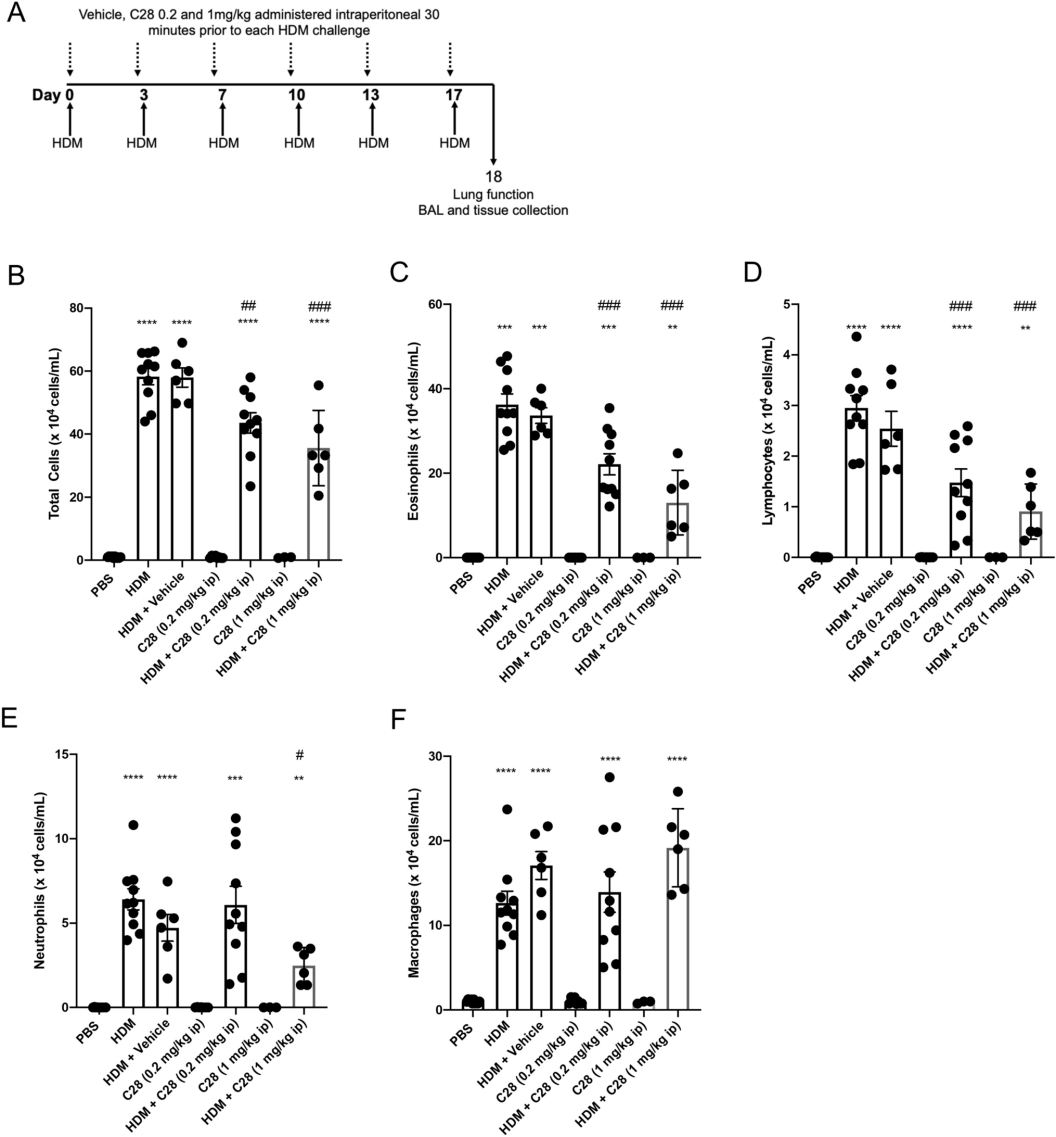
C28 at low and high doses inhibits airway inflammation in HDM-induced experimental asthma. **A** Mice received either PBS or HDM extract twice a week for 3 weeks. Mice were treated either with C28 at low (0.2 mg/kg) or high (1 mg/kg) dose intraperitoneally 30 min prior to each HDM instillation. Control mice were treated with PBS only. The number of total cells (**B**), eosinophils (**C**), lymphocytes (**D**), neutrophils (**E**) and macrophages (**F**) in the bronchoalveolar lavage fluid were quantified. Data represent mean values ± SEM. **P ≤ 0.01, ***P ≤ 0.001 and ****P ≤ 0.0001 versus the PBS group. #*P* ≤ 0.05, ##*P* ≤ 0.01 and ###*P* ≤ 0.001 versus the HDM group. N = 6–10 mice per group. HDM = house dust mite

To further assess the efficacy of C28 against HDM-induced detrimental features of asthma, we performed experiments in which C28 was administered at both a low (0.2 mg/kg) and high (1 mg/kg) dose intraperitoneally, 30 min before each HDM challenge (see Fig. 1A). This showed that treatment with C28 at both low- and high dose attenuated HDM-induced airway inflammation, as shown by significantly reduced BAL infiltration of total cells, eosinophils, lymphocytes and neutrophils (Fig. 1B–E). In contrast, no change in macrophage numbers was observed in response to high- or low dose C28 treatment (Fig. 1F).

**Fig. 2:**
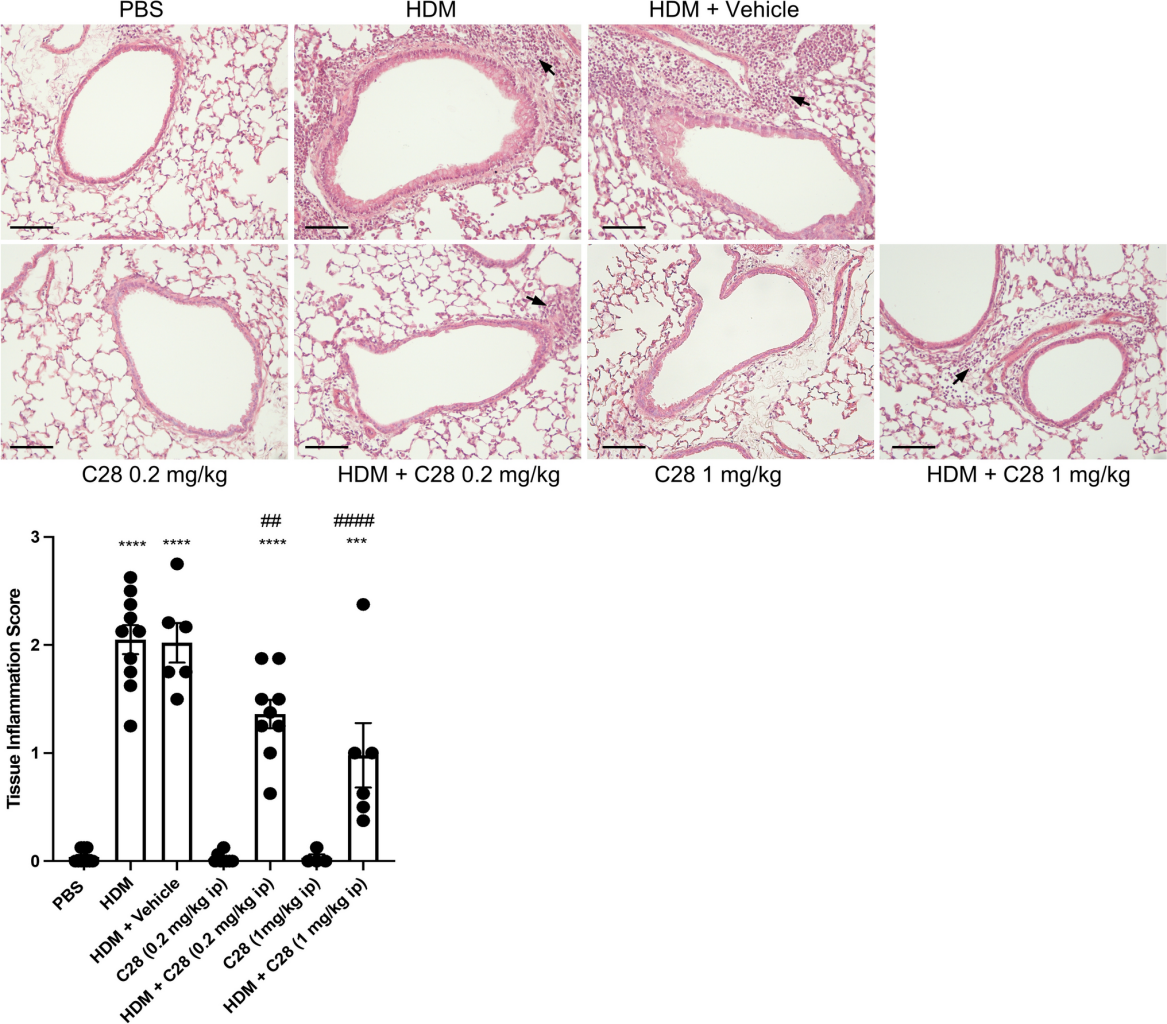
C28 at low and high doses inhibits tissue inflammation in HDM-induced experimental asthma. Mice received either PBS or HDM extract twice a week for 3 weeks. Mice were treated either with C28 at low (0.2 mg/kg) or high (1 mg/kg) dose intraperitoneally 30 min prior to each HDM instillation. Control mice were treated with PBS only. Tissue inflammation was assessed after staining of lung sections with hematoxylin and eosin (H&E). Arrows indicate leukocyte infiltrates. Scale bars: 100 µm. Images of the respective whole lung sections are shown in Supplementary Fig. 5A. Quantification of tissue inflammation by scoring is shown below. Data represent mean values ± SEM. ***P ≤ 0.001 and ****P ≤ 0.0001 versus the PBS group. ##*P* ≤ 0.01 and ####*P* ≤ 0.0001 versus the HDM group. N = 6–10 mice per group. HDM = house dust mite

**Fig. 3:**
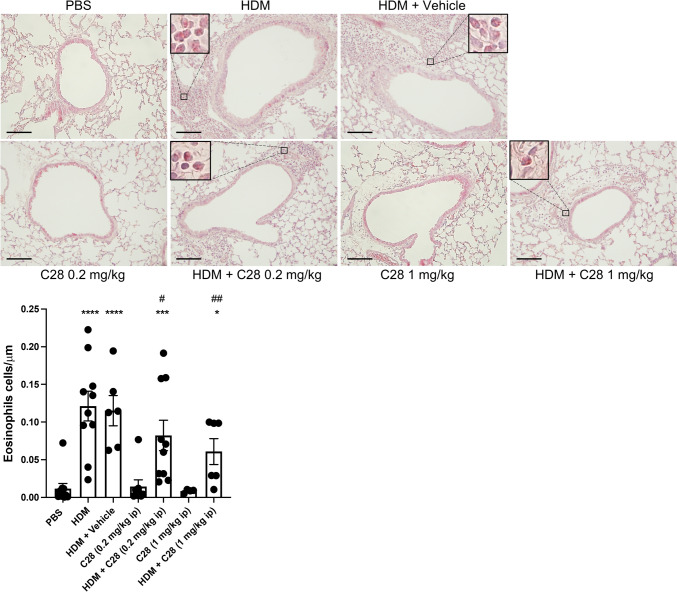
C28 at both low and high doses inhibits tissue eosinophil inflammation in HDM-induced experimental asthma. Mice received either PBS or HDM extract twice a week for 3 weeks. Mice were treated either with C28 at low (0.2 mg/kg) or high (1 mg/kg) dose intraperitoneally 30 min prior to each HDM instillation. Control mice were treated with PBS only. Tissue eosinophilia was assessed after staining of lung sections with chromotrope 2R. Eosinophils are shown in inserts with higher magnifications. Scale bars: 100 µm. Images of the respective whole lung sections are shown in Supplementary Fig. 5B. Quantification of eosinophils is shown below. Data represent mean values ± SEM. *P ≤ 0.05, ***P ≤ 0.001 and ****P ≤ 0.0001 versus the PBS group. #*P* ≤ 0.05 and ##*P* ≤ 0.01 versus the HDM group. N = 6–10 mice per group. HDM = house dust mite

To further assess the anti-inflammatory effects of C28, we measured the tissue inflammation around the airways and enumerated eosinophils. As seen in Fig. 2, a significant elevation of tissue inflammation was seen in the HDM-challenged versus control mice. Further, treatment of mice with C28, at both low and high doses, attenuated the HDM-induced tissue inflammation around the airways (Fig. 2). This was accompanied by reduced tissue eosinophilia (Fig. 3).

### C28 protects against HDM-induced airway hyperresponsiveness and goblet hyperplasia

**Fig. 4:**
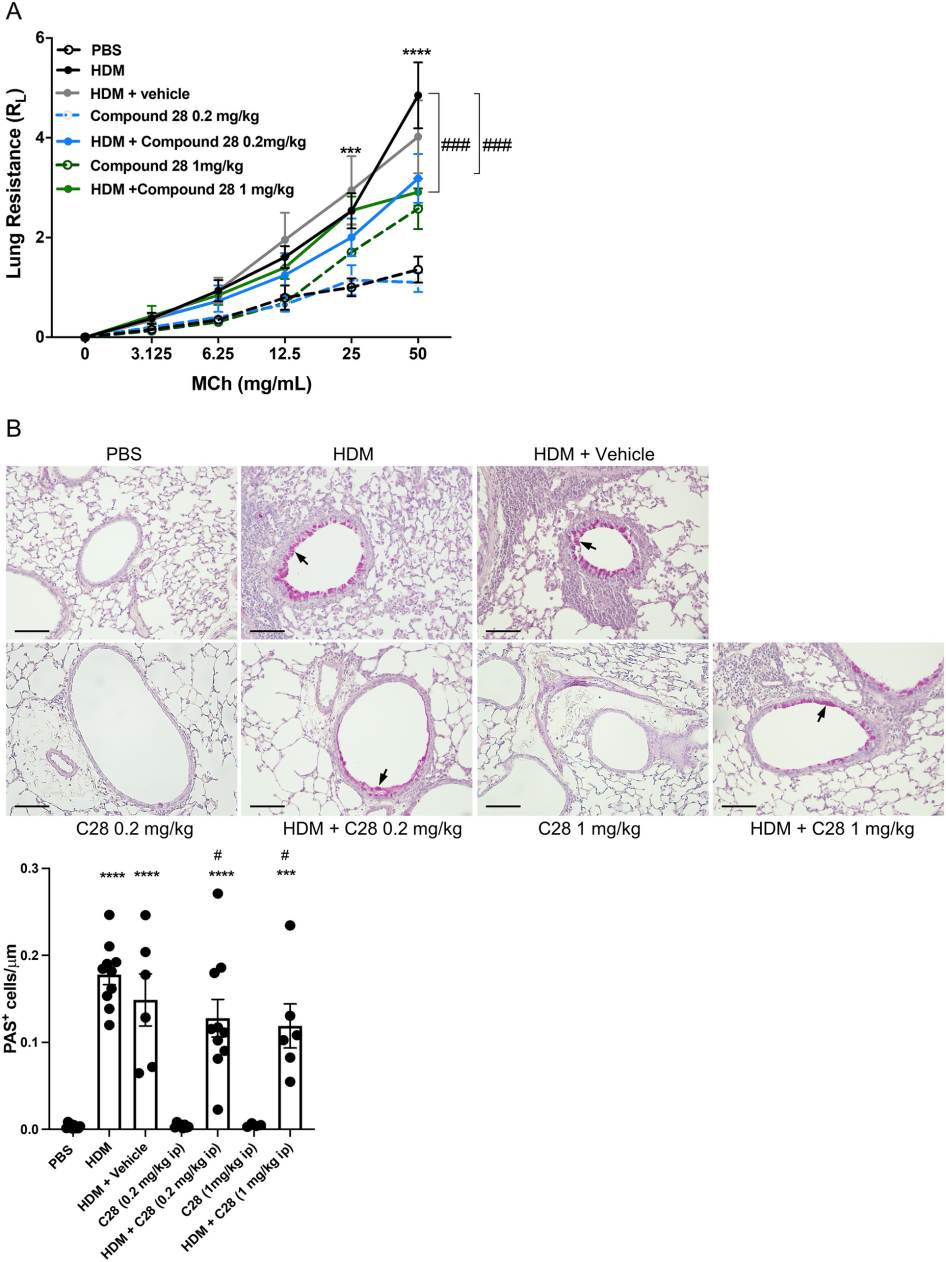
C28 at both low and high doses inhibits AHR and goblet hyperplasia in HDM-induced experimental asthma. Mice received either PBS or HDM extract twice a week for 3 weeks. Mice were treated either with C28 low (0.2 mg/kg) or high (1 mg/kg) dose intraperitoneally 30 min prior to each HDM instillation. Control mice were treated with PBS only. **A** Lung resistance (R_L_) was measured using a Buxco FinePointe series instrument. **B** Goblet hyperplasia around the airways in the lung sections was assessed by PAS staining. Arrows indicate PAS^+^ cells (goblet cells). Scale bars: 100 µm. Images of the respective whole lung sections are shown in Supplementary Fig. 6A. Quantification of PAS^+^ cells is shown below. Data represent mean values ± SEM. ***P ≤ 0.001 and ****P ≤ 0.0001 versus the PBS group. #*P* ≤ 0.05 and ###*P* ≤ 0.001 versus the HDM group. N = 6–10 mice per group. HDM = house dust mite

We next assessed whether low- and/or high doses of C28 can suppress HDM-induced AHR (in response to methacholine). As expected, challenge of mice with HDM alone induced a significant rise in R_L_ as compared with vehicle controls (Fig. 4A). In contrast, no effects of HDM sensitization on dynamic compliance (Cdyn) were seen (Suppl Fig. 4B). Notably, the HDM-induced AHR was significantly mitigated by treating the mice with either low- or high doses of C28, whereas vehicle alone had no effect (Fig. 4A). Moreover, administration of C28 alone did not affect baseline reactivity to methacholine, although a non-significant trend of increased reactivity against methacholine was observed in mice given the higher dose of C28.

We also examined whether C28 could affect goblet cell hyperplasia, as assessed by PAS staining of histological sections, followed by the quantification of PAS^+^ cells in the airway epithelia. These assessments revealed a significant goblet cell hyperplasia in response to HDM challenge, which was mitigated by administration of C28 at both the low- and high dose, but not by administration of vehicle alone (Fig. 4B).

### High-dose C28 prevents HDM-induced thickening of airway smooth muscle layer

**Fig. 5:**
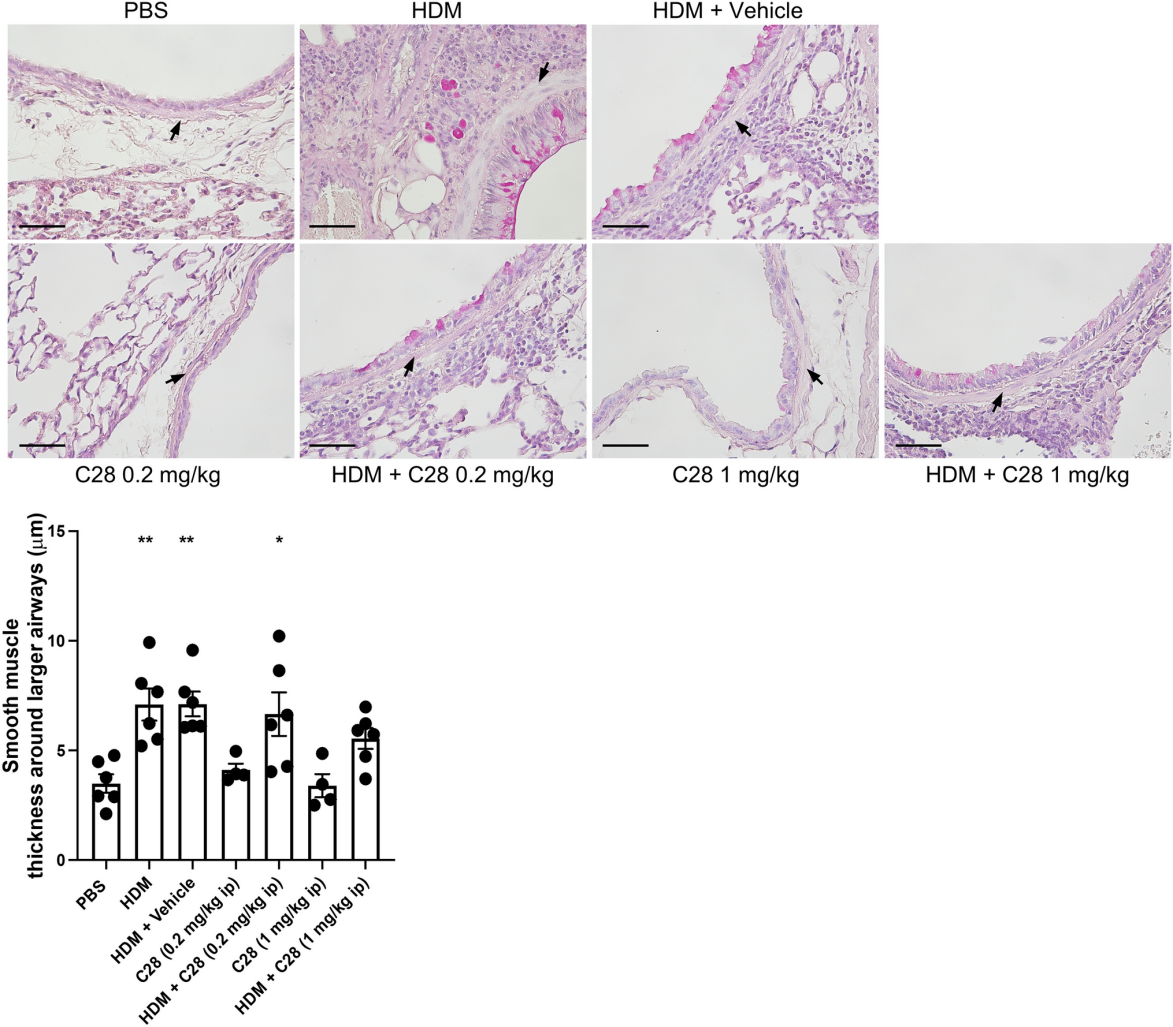
C28 at high dose suppresses smooth muscle cell proliferation in HDM-induced experimental asthma. Mice received either PBS or HDM extract twice a week for 3 weeks. Mice were treated either with C28 low (0.2 mg/kg) or high (1 mg/kg) dose intraperitoneally 30 min prior to each HDM instillation. Control mice were treated with PBS only. Smooth muscle cell proliferation around the airways was assessed by PAS staining. Arrows indicate smooth muscle layer around larger airways. Scale bars: 50 µm. Images of the respective whole lung sections are shown in Supplementary Fig. 6B. Data represent mean values ± SEM. *P ≤ 0.05, and **P ≤ 0.01 versus the PBS group. N = 4–6 mice per group. HDM = house dust mite

Smooth muscle proliferation and thickening of the airway smooth muscle layer is a characteristic feature of asthma, which promotes airway remodeling and AHR. As seen in Fig. 5, thickening of the smooth muscle layer around the airways was evident in HDM-challenged mice that were either untreated, treated with low dose C28 (0.2 mg/kg), or given only vehicle. In contrast, a blockade of HDM-induced thickening of the smooth muscle layer was seen upon treatment with the high dose of C28 (1 mg/kg) (Fig. 5).

### C28 mitigates HDM-induced inflammatory gene expression in the lungs

**Fig. 6:**
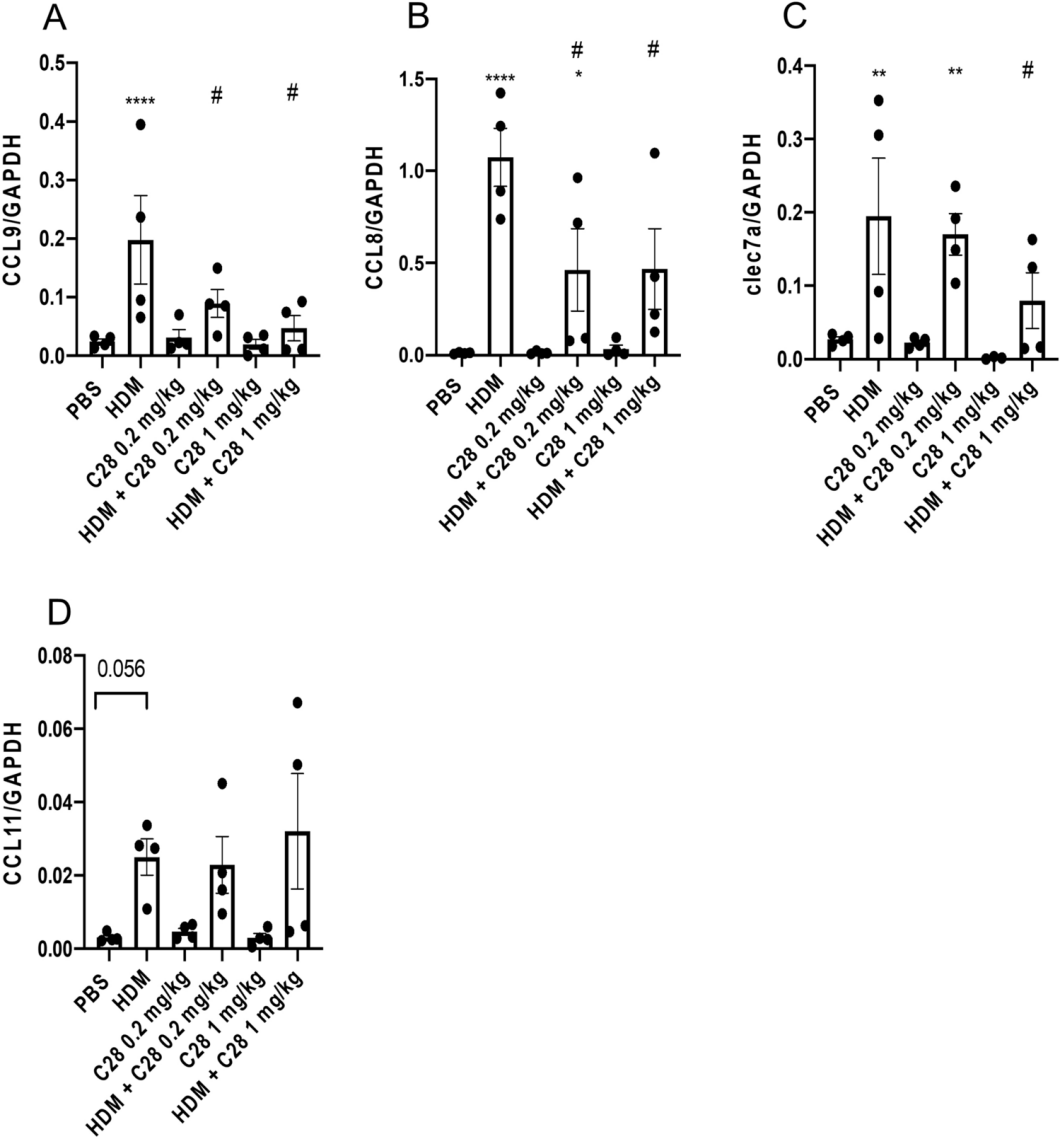
C28 suppresses the expression of the Ccl8 and Ccl9 and Clec7a/Dectin1 genes in HDM-induced experimental asthma. Mice received either PBS or HDM extract twice a week for 3 weeks. Mice were treated either with C28 low (0.2 mg/kg) or high (1 mg/kg) dose intraperitoneally 30 min prior to each HDM instillation. Control mice were treated with PBS only. Total RNA was recovered from the lungs and was assessed by qPCR analysis for levels of mRNA coding for: Ccl9 (**A**), Ccl8 (**B**), Clec7a/Dectin1 (**C**), Ccl11 (**D**). Data was normalized to GAPDH expression. Data represent mean values ± SEM. *P ≤ 0.05, **P ≤ 0.01, and ****P ≤ 0.0001 versus the PBS group. #*P* ≤ 0.05 and ##*P* ≤ 0.01 versus the HDM group. N = 4 mice per group. HDM = house dust mite

In a previous study we showed that sensitization of mice to HDM could induce the expression of genes coding for various inflammatory markers, including Ccl8, Ccl9, Ccl11 and Clec7a/Dectin1 [22]. To explore whether the observed anti-inflammatory effects of C28 are reflected by effects on inflammatory gene expression, we assessed whether C28 treatment could affect the expression of these genes. In agreement with previous findings, HDM sensitization induced the expression of all these inflammatory markers, i.e., Ccl8, Ccl9, Ccl11 and Clec7a/Dectin1 (Fig. 6A–D). Furthermore, treatment of HDM-sensitized mice with C28 at both the low and high dose significantly attenuated the expression of Ccl9 (Fig. 6A) and Ccl8 (Fig. 6B). Clec7a/Dectin1 expression was also reduced, although only in mice treated with the high dose of C28 (Fig. 6C). In contrast, although an increased expression of Ccl11 was noticed in response to HDM, no reduction in its expression was observed in mice treated with C28, irrespective of the dose (Fig. 6D).

### C28 inhibits the MCP activity in both in vivo and ex vivo conditions

Collectively, the results above indicate that C28 has ameliorating effects on various manifestations of HDM-induced allergic lung inflammation, suggesting that enzymes of MCP family can have a detrimental role in such settings. In the next set of experiments, the aim was to approach the nature of such asthma-promoting MCP activity, by assessing the ability of C28 to inhibit different types of MCPs in a lung- and plasma context. To this end, we employed two different conditions: either in vivo by analyzing lungs or plasma collected from naïve mice injected i.p. with C28 (1 mg/kg), or ex vivo by adding increasing concentrations of C28 (0.1, 1 or 10 nM) to lung extracts collected from naïve or HDM-challenged mice. In both cases, exogenous CPA1 (as a representative for CPA-type MCPs), CPB1 (as a representative for CPB-type MCPs) or ACE2 (implicated in lung inflammatory responses) was added to the lung extracts or plasma, followed by measurements of residual MCP activities. These analyses revealed that in vivo treatment with C28 translated into efficient inhibition of CPA1 within lung extracts, whereas a lower extent of CPB1 and ACE2 inhibition was seen (Table 2). Further, efficient inhibition of all three MCPs, in particular CPA1, was seen in the plasma context (Table 2).

**Table 2:** Relative CPA1, CPB and ACE2 activity of exogenous MCPs added to lung homogenates and plasma from mice treated with C28 (untreated conditions were used as 100%)^a^

Condition	CPA1 activity (%)	CPB activity (%)	ACE2 activity (%)
Untreated lungs	100.0 ± 0.7	100.0 ± 0.8	100.0 ± 0.8
Lungs C28	2.8 ± 0.4	41.7 ± 2.5	75.4 ± 1.4
Untreated Plasma	100% ± 0.4	100% ± 0.7	100% ± 1.2
Plasma C28	1.1 ± 0.2	6.3% ± 1.3	12.4% ± 4.53.8

^a^Blood and lungs were collected thirty minutes after intraperitoneal injection of naïve mice with C28 (1 mg/kg) or from untreated naïve mice as controls. Activity values are represented as percentage of the untreated condition. All samples were measured in triplicates

To further assess the possible impact of C28 on CPB-type MCPs and ACE2, a dose response experiment was performed. This revealed a dose dependent inhibition of CPB1 in lung extracts to which C28 had been added (Table 3).

**Table 3:** Relative CPB activity of exogenous porcine CPB1 added to lung homogenates from control and asthmatic mice in presence of C28, at increasing concentrations^a^

Condition	Untreated (%)	0.1 nM C28 (%)	1 nM C28 (%)	10 nM C28 (%)
Control mice	100.0 ± 2.0	83.6 ± 2.2	34.2 ± 3.3	14.2 ± 2.3
Asthmatic mice	100.0 ± 2.7	84.7 ± 5.5	48.6 ± 4.6	22.7 ± 2.0

^a^Activity values are represented as percentage of the respective untreated condition. All samples were measured in triplicates

Notably, CPB1 inhibition was seen both in extracts from control- and HDM-sensitized mice (Table 3). In contrast, only a weak inhibition of ACE2 activity by C28 was seen when using this approach, irrespective of the C28 concentration (Table 4). Together, these findings suggest that CPA-like- and CPB-like MCPs may be the primary targets for C28 to explain its dampening effects on allergic lung inflammation.

**Table 4:** Relative ACE2 activity of exogenous mouse ACE2 added to lung homogenates from control and asthmatic mice in presence of C28, at increasing concentrations^a^

Condition	Untreated (%)	0.1 nM C28 (%)	1 nM C28 (%)	10 nM C28 (%)
Control mice	100.0 ± 1.6	95.3 ± 3.0	94.7 ± 4.1	86.5 ± 4.3
Asthmatic mice	100.0 ± 2.5	100.9 ± 2.1	97.7 ± 1.6	87.3 ± 2.2

^a^Activity values are represented as percentage of the respective untreated condition. All samples were measured in triplicates

## Discussion

MCPs of the M14A family are implicated in a range of both physiological and pathological contexts [24, 25]. Based on this, inhibition of such enzymes is currently considered for possible therapeutic purposes. Accordingly, several low molecular weight MCP inhibitors have been developed [26, 27]. In the present study, we evaluated two such inhibitors, C8 [21] and C28 [20] for their possible impact in a mouse model for allergic asthma. Notably, we used an asthma model that is of high physiological relevance, being based on one of the major allergens responsible for the development of human asthma—house dust mite (HDM)[28]. Hence, it is therefore reasonable to assume that the findings generated in this mouse model setting may be translated into human, corresponding conditions.

Our findings reveal that one of the tested compounds, C28, had strong anti-inflammatory properties. This was, firstly, manifested as an inhibitory impact on the infiltration of several inflammatory cells into the BAL fluid of mice sensitized and challenges with HDM extract. Importantly, a marked decrease was seen in the infiltration of eosinophils, these cells representing major detrimental cell populations in allergic asthma [29]. In addition, a reduced lymphocyte and neutrophil infiltration was also seen, whereas macrophage infiltration into the BAL fluid was not affected. Importantly, the anti-inflammatory effects of C28 were also translated into effects on the actual lung tissue, in which markedly lower tissue inflammation was seen after treatment of mice with C28. As for the BAL fluid, a marked reduction of eosinophil infiltration into the lung tissue was seen in response to C28. Hence, C28 has a strong capacity to block inflammatory cell accumulation in this model of allergic lung inflammation, and we also noted that this capacity was reflected by a dampening impact on the expression of important pro-inflammatory chemokines and also on Clec7a, the latter being a cell surface receptor which has been implicated as key factor in the development of asthma [30].

In addition to these effects on inflammatory cells, we show that C28 has suppressive effects on goblet cell populations, implying that C28 can relieve mucus production under asthmatic conditions. Another important finding was that C28 suppresses the airway responsiveness to methacholine in HDM-sensitized/challenged animals. AHR is a hallmark event in allergic asthma, and the ability of C28 to interfere with this symptom is hence a critical finding. In agreement with its dampening effects on AHR, we demonstrate that C28 (at the higher used dose) blocked the thickening of the airway smooth muscle cell layer that is seen in response to HDM sensitization. Together, our findings thus show that C28 suppresses a range of cardinal features related to allergic airway inflammation, and these findings may thus encourage future initiatives to develop this compound for therapeutic purposes in allergic asthma.

The findings reported here are clearly in line with a previous study, in which we showed that a natural MCP inhibitor purified from *Nerita versicolor*, NvCI, suppressed multiple inflammatory features in a mouse model for allergic asthma [18]. However, NvCI is a protein, and is therefore not suitable as a therapeutic drug. In contrast, C28 is a low molecular weight, synthetic compound soluble in PBS, and can due to these properties be a candidate for usage as a therapeutic. An important finding in this study was that, after i.p. administration (in vivo), C28 reached the lung tissue in active form, being able to strongly inhibit CPA-like and to some extent CPB-like MCPs. Hence, C28 appears to have properties that are highly compatible with usage as a systemic drug capable of blocking CPA-like and CPB-like activity.

Although the present findings show that MCP inhibition has a capacity to block hallmark features of allergic asthma, we are at present not able to pinpoint the exact molecular target(s) for C28 under these conditions. Our initial hypothesis was that mast cell CPA3 might represent a target, based on the implication of CPA3 in human asthma [7, 31, 32]. However, we showed recently that the absence of CPA3 did not influence the outcome in a mouse model of asthma, arguing against a major role for CPA3 in regulating allergic asthma [19]. On the other hand, we cannot exclude that CPA3, despite its redundancy in the mouse asthma model, might have an impact on asthmatic settings in humans. ACE2 is a MCP belonging to the M2 family that has previously been implicated in inflammatory lung conditions such as COVID-19 [15] and severe asthma [16], and we thus considered the possibility that ACE2 could contribute to the symptoms seen in the HDM-asthma model. However, we noted only weak inhibition of ACE2 by C28 under in vivo and ex vivo conditions, arguing against that ACE2 is a major significant target for C28. In contrast, more efficient inhibition of CPA- and CPB-like MCPs was observed. We may thus propose that such MCPs may represent targets for C28 in HDM-induced asthma. Out of the two assessed MCP inhibitors (C8 and C28) it has previously been shown that C8 is a potent inhibitor of CPA-like MCPs but has no inhibitory impact on CPB [20], which was also confirmed in the present study (Table 1). In contrast, we found that C28 is a potent inhibitor of both CPA- and CPB-like enzymes. Based on this, we may propose that MCPs with CPB-like properties are major targets for C28 in the HDM-asthma setting, at least by now.

The exact mechanism behind the anti-asthmatic effects of C28 are intriguing, a key question being to identify the putative MCP(s) having pro-inflammatory properties in the HDM-asthma context. Previous studies have shown that MCPs in fact can have anti-inflammatory activity, by cleaving the C3a and C5a anaphylatoxins [33]. In other studies, it has been shown that MCPs (TAFI) can have anti-fibrinolytic activity, implying that its inhibition could lead to excessive fibrin deposition, thereby contributing to asthma-associated tissue remodeling. In line with the latter, elevation of TAFI levels has been seen in asthma [10, 11, 34]. On the other hand, TAFI has been reported to have protective functions in experimental asthma [13], and it thus appears unlikely that effects of C28 on TAFI can explain the anti-inflammatory effects of this compound in the HDM-asthma model. Another possibility is that MCP inhibition could suppress asthma by reducing the availability of arginine for NO synthesis [35]. However, more detailed in investigations are warranted to identify the exact molecular mechanism explaining the ameliorating effects of C28 on the outcome in HDM-induced allergic lung inflammation.

In conclusion, our study shows that C28, an inhibitor of A/B-type carboxypeptidases, can suppress multiple asthma-like symptoms in a physiologically relevant mouse model. Moreover, C28 was shown to reach the lungs in active form and could strongly inhibit both CPA and CPB-like activity. Thus, targeting A/B-type carboxypeptidases holds great promise as a potential regimen in treatment of allergic asthma.

## Supplementary Information

Below is the link to the electronic supplementary material.

Supplementary file1 (PDF 3423 KB)

## Data Availability

Data will be made available on request.

## Abbreviations

HDM: House dust mite
AHR: Airway hyperresponsiveness
BAL: Bronchoalveolar lavage
PAS: Periodic acid-Schiff
MCP: Metallo-carboxypeptidase
CP: Carboxypeptidase
TAFI: Thrombin-activatable fibrinolysis inhibitor
ACE: Angiotensin-converting enzyme

## References

[1] Mukherjee AB, Zhang Z. Allergic asthma: influence of genetic and environmental factors. J Biol Chem. 2011;286:32883–9. 21799018 10.1074/jbc.R110.197046 PMC3190897

[2] Akula S, Hellman L, Avilés FX, Wernersson S. Analysis of the mast cell expressed carboxypeptidase A3 and its structural and evolutionary relationship to other vertebrate carboxypeptidases. Dev Comp Immunol. 2022;127: 104273. 34619175 10.1016/j.dci.2021.104273

[3] Tanco S, Lorenzo J, Garcia-Pardo J, Degroeve S, Martens L, Aviles FX, et al. Proteome-derived peptide libraries to study the substrate specificity profiles of carboxypeptidases. Mol Cell Proteomics. 2013;12:2096–110. 23620545 10.1074/mcp.M112.023234 PMC3734572

[4] Arolas JL, Gomis-Rüth FX. Zinc metallocarboxypeptidases. In: Kretsinger RH, Uversky VN, Permyakov EA, editors. Encyclopedia of metalloproteins. New York: Springer; 2013. p. 2473–9.

[5] Arolas JL, Vendrell J, Aviles FX, Fricker LD. Metallocarboxypeptidases: emerging drug targets in biomedicine. Curr Pharm Des. 2007;13:349–66. 17311554 10.2174/138161207780162980

[6] Dougherty RH, Sidhu SS, Raman K, Solon M, Solberg OD, Caughey GH, et al. Accumulation of intraepithelial mast cells with a unique protease phenotype in T(H)2-high asthma. J Allergy Clin Immunol. 2010;125:1046-1053.e8. 20451039 10.1016/j.jaci.2010.03.003 PMC2918406

[7] Fricker M, Gibson PG, Powell H, Simpson JL, Yang IA, Upham JW, et al. A sputum 6-gene signature predicts future exacerbations of poorly controlled asthma. J Allergy Clin Immunol. 2019;144:51-60.e11. 30682452 10.1016/j.jaci.2018.12.1020

[8] Winter NA, Qin L, Gibson PG, McDonald VM, Baines KJ, Faulkner J, et al. Sputum mast cell/basophil gene expression relates to inflammatory and clinical features of severe asthma. J Allergy Clin Immunol. 2021;148:428–38. 33609626 10.1016/j.jaci.2021.01.033

[9] Siddhuraj P, Jönsson J, Alyamani M, Prabhala P, Magnusson M, Lindstedt S, Erjefält JS. Dynamically upregulated mast cell CPA3 patterns in chronic obstructive pulmonary disease and idiopathic pulmonary fibrosis. Front Immunol. 2022;13: 924244. 35983043 10.3389/fimmu.2022.924244 PMC9378779

[10] Kemona-Chetnik I, Kowal K, Kucharewicz I, Pampuch A. Bodzenta-Lukaszyk A [Thrombin activatable fibrinolysis inhibitor (TAFI) in allergic asthma patients]. Przegl Lek. 2006;63:1281–5. 17642140

[11] Imoto Y, Kato A, Takabayashi T, Stevens W, Norton JE, Suh LA, et al. Increased thrombin-activatable fibrinolysis inhibitor levels in patients with chronic rhinosinusitis with nasal polyps. J Allergy Clin Immunol. 2019;144:1566-1574.e6. 31562871 10.1016/j.jaci.2019.08.040 PMC6900453

[12] Naito M, Taguchi O, Kobayashi T, Takagi T, D’Alessandro-Gabazza CN, Matsushima Y, et al. Thrombin-activatable fibrinolysis inhibitor protects against acute lung injury by inhibiting the complement system. Am J Respir Cell Mol Biol. 2013;49:646–53. 23721130 10.1165/rcmb.2012-0454OC

[13] Fujiwara A, Taguchi O, Takagi T, D’Alessandro-Gabazza CN, Boveda-Ruiz D, Toda M, et al. Role of thrombin-activatable fibrinolysis inhibitor in allergic bronchial asthma. Lung. 2012;190:189–98. 22037793 10.1007/s00408-011-9337-9

[14] Sanglas L, Arolas JL, Valnickova Z, Aviles FX, Enghild JJ, Gomis-Rüth FX. Insights into the molecular inactivation mechanism of human activated thrombin-activatable fibrinolysis inhibitor. J Thromb Haemost. 2010;8:1056–65. 20088943 10.1111/j.1538-7836.2010.03740.x

[15] Reindl-Schwaighofer R, Hödlmoser S, Eskandary F, Poglitsch M, Bonderman D, Strassl R, et al. ACE2 Elevation in Severe COVID-19. Am J Respir Crit Care Med. 2021;203:1191–6. 33600742 10.1164/rccm.202101-0142LE PMC8314901

[16] Kermani NZ, Song W-J, Badi Y, Versi A, Guo Y, Sun K, et al. Sputum ACE2, TMPRSS2 and FURIN gene expression in severe neutrophilic asthma. Respir Res. 2021;22:10. 33413387 10.1186/s12931-020-01605-8 PMC7788167

[17] Covaleda-Cortés G, Hernández M, Trejo SA, Mansur M, Rodríguez-Calado S, García-Pardo J, et al. Characterization, recombinant production and structure-function analysis of NvCI, a picomolar metallocarboxypeptidase inhibitor from the marine snail Nerita versicolor. Mar Drugs. 2019;17:511. 31470614 10.3390/md17090511 PMC6780499

[18] Waern I, Taha S, Lorenzo J, Montpeyó D, Covaleda-Cortés G, Avilés FX, Wernersson S. Carboxypeptidase inhibition by NvCI suppresses airway hyperreactivity in a mouse asthma model. Allergy. 2021;76:2234–7. 33387397 10.1111/all.14730

[19] Waern I, Akula S, Allam VSRR, Taha S, Feyerabend TB, Åbrink M, Wernersson S. Disruption of the mast cell carboxypeptidase A3 gene does not attenuate airway inflammation and hyperresponsiveness in two mouse models of asthma. PLoS ONE. 2024;19: e0300668. 38578780 10.1371/journal.pone.0300668 PMC10997103

[20] Mores A, Matziari M, Beau F, Cuniasse P, Yiotakis A, Dive V. Development of potent and selective phosphinic peptide inhibitors of angiotensin-converting enzyme 2. J Med Chem. 2008;51:2216–26. 18324760 10.1021/jm701275z

[21] Covaleda G, Gallego P, Vendrell J, Georgiadis D, Lorenzo J, Dive V, et al. Synthesis and structural/functional characterization of selective M14 metallocarboxypeptidase inhibitors based on phosphinic pseudopeptide scaffold: implications on the design of specific optical probes. J Med Chem. 2019;62:1917–31. 30688452 10.1021/acs.jmedchem.8b01465

[22] Allam V, Waern I, Taha S, Akula S, Wernersson S, Pejler G. Nafamostat has anti-asthmatic effects associated with suppressed pro-inflammatory gene expression, eosinophil infiltration and airway hyperreactivity. Front Immunol. 2023;14:1136780. 37153590 10.3389/fimmu.2023.1136780 PMC10160450

[23] Waern I, Jonasson S, Hjoberg J, Bucht A, Åbrink M, Pejler G, Wernersson S. Mouse mast cell protease 4 is the major Chymase in murine airways and has a protective role in allergic airway inflammation. J Immunol. 2009;183:6369. 19841188 10.4049/jimmunol.0900180

[24] Gomis-Rüth FX. Structure and mechanism of metallocarboxypeptidases. Crit Rev Biochem Mol Biol. 2008;43:319–45. 18937105 10.1080/10409230802376375

[25] Otero A, de la Vega MR, Tanco S, Lorenzo J, Avilés FX, Reverter D. The novel structure of a cytosolic M14 metallocarboxypeptidase (CCP) from Pseudomonas aeruginosa: a model for mammalian CCPs. FASEB J. 2012;26:3754–64. 22645247 10.1096/fj.12-209601

[26] Fernández D, Pallarès I, Covaleda G, Avilés FX, Vendrell J. Metallocarboxypeptidases and their inhibitors: recent developments in biomedically relevant protein and organic ligands. Curr Med Chem. 2013;20:1595–608. 23432588 10.2174/0929867311320120009

[27] Fernández D, Pallarès I, Vendrell J, Avilés FX. Progress in metallocarboxypeptidases and their small molecular weight inhibitors. Biochimie. 2010;92:1484–500. 20466032 10.1016/j.biochi.2010.05.002

[28] Phillips JE, Peng R, Harris P, Burns L, Renteria L, Lundblad LKA, et al. House dust mite models: will they translate clinically as a superior model of asthma? J Allergy Clin Immunol. 2013;132:242–4. 23403050 10.1016/j.jaci.2012.12.1571

[29] Possa SS, Leick EA, Prado CM, Martins MA, Tibério IF. Eosinophilic inflammation in allergic asthma. Front Pharmacol. 2013;4:46. 23616768 10.3389/fphar.2013.00046 PMC3627984

[30] Ito T, Hirose K, Norimoto A, Tamachi T, Yokota M, Saku A, et al. Dectin-1 plays an important role in house dust mite-induced allergic airway inflammation through the activation of CD11b+ dendritic cells. J Immunol. 2017;198:61–70. 27852745 10.4049/jimmunol.1502393

[31] Sverrild A, Bergqvist A, Baines KJ, Porsbjerg C, Andersson CK, Thomsen SF, et al. Airway responsiveness to mannitol in asthma is associated with chymase-positive mast cells and eosinophilic airway inflammation. Clin Exp Allergy. 2016;46:288–97. 26252943 10.1111/cea.12609

[32] Yan Z, Liu L, Jiao L, Wen X, Liu J, Wang N. Bioinformatics analysis and identification of underlying biomarkers potentially linking allergic rhinitis and asthma. Med Sci Monit. 2020;26: e924934. 32460303 10.12659/MSM.924934 PMC7278529

[33] Leung LLK, Morser J. Carboxypeptidase B2 and carboxypeptidase N in the crosstalk between coagulation, thrombosis, inflammation, and innate immunity. J Thromb Haemost. 2018;16:1474–86. 10.1111/jth.14199 29883024

[34] Brims FJ, Chauhan AJ, Higgins B, Shute JK. Coagulation factors in the airways in moderate and severe asthma and the effect of inhaled steroids. Thorax. 2009;64:1037–43. 19703828 10.1136/thx.2009.114439

[35] Hadkar V, Skidgel RA. Carboxypeptidase D is up-regulated in raw 264.7 macrophages and stimulates nitric oxide synthesis by cells in arginine-free medium. Mol Pharmacol. 2001;59:1324–32. 11306718 10.1124/mol.59.5.1324

