# Supplementary material for: Targeting carboxypeptidase A/B activity with the phosphinic inhibitor C28 reduces the asthmatic response in a mouse model of house dust mite-induced asthma
Source: Inflamm Res. 2025 May 24;74(1):80. doi: 10.1007/s00011-025-02046-z (PMC12103337; doi:10.1007/s00011-025-02046-z)
Supplement: Supplementary file 1 — Supplementary file1 (PDF 3423 KB) [file 11_2025_2046_MOESM1_ESM.pdf]

Targeting carboxypeptidase A/B activity with the phosphinic inhibitor C28 reduces the asthmatic response in a mouse model of house dust mite-induced asthma

Journal: Inflammation Research

Authors:
Venkata Sita Rama Raju Allam[1, 2], David Montpeyó[3], Fabrice Beau[5], Sowsan Taha[1], Ida Waern[1], Srinivas Akula[1,2], Francesc Xavier Avilés[3], Julia Lorenzo[3,4], Laurent Devel[5], Gunnar Pejler[2], Sara Wernersson[1]

1 Department of Animal Biosciences, Swedish University of Agricultural Sciences, Uppsala, Sweden.

2 Department of Medical Biochemistry and Microbiology, Uppsala University, Uppsala, Sweden.

3 Institut de Biotecnologia i de Biomedicina (IBB) and Departament de Bioquímica i de Biologia Molecular, Universitat Autònoma de Barcelona, Bellaterra, Barcelona, Spain.

4 Centro de Investigación Biomédica en Red, Bioingeniería, Biomateriales y Nanomedicina (CIBER-BBN), 08193 Cerdanyola del Vallès, Spain.

5 CEA, INRAE, Médicaments et Technologies pour la Santé (MTS), SIMoS, Université Paris-Saclay, 91191, Gif-sur-Yvette, (France)

Address correspondence to: Sara Wernersson, Swedish University of Agricultural Sciences, Department of Animal Biosciences, Uppsala Sweden; sara.wernersson@slu.se or Venkata Sita Rama Raju Allam, Uppsala University, Department of Medical Biochemistry and Microbiology, Uppsala, Sweden; venkata.allam@imbim.uu.se

Suppl Fig 1

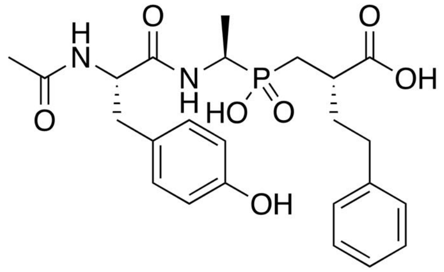

C8

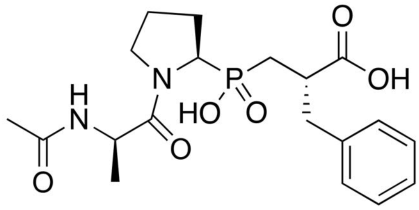

C28

Suppl Fig 2

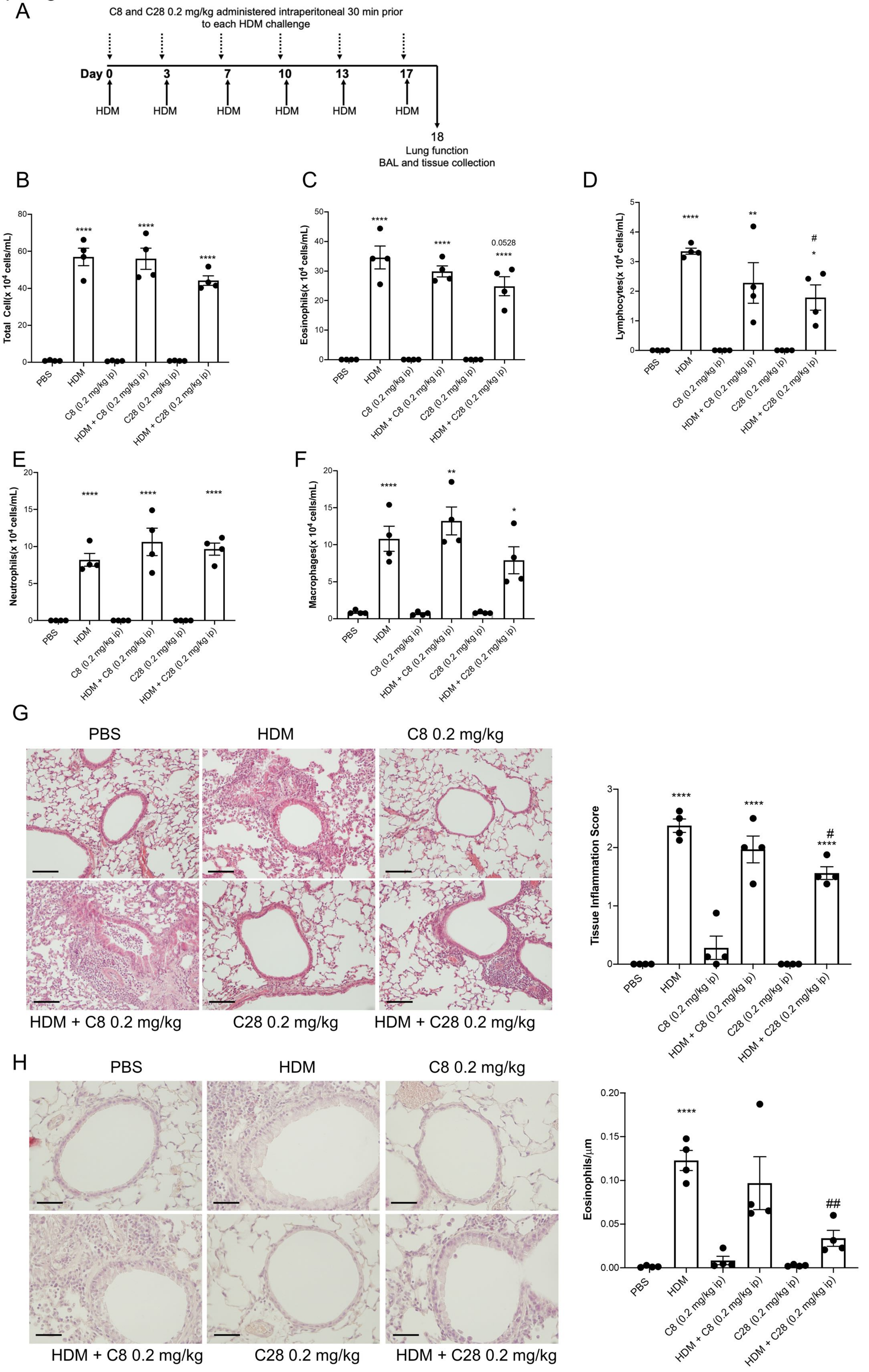
A
C8 and C28 0.2 mg/kg administered intraperitoneal 30 min prior to each HDM challenge
Day 0
3
7
10
13
17
HDM
18
Lung function
BAL and tissue collection
B
Total Cell(x 10^4 cells/mL)
C
Eosinophils(x 10^4 cells/mL)
0.0528
D
Lymphocytes(x 10^4 cells/mL)
E
Neutrophils(x 10^4 cells/mL)
F
Macrophages(x 10^4 cells/mL)
PBS
HDM
C8 (0.2 mg/kg ip)
HDM + C8 (0.2 mg/kg ip)
C28 (0.2 mg/kg ip)
HDM + C28 (0.2 mg/kg ip)
G
PBS
HDM
C8 0.2 mg/kg
HDM + C8 0.2 mg/kg
C28 0.2 mg/kg
HDM + C28 0.2 mg/kg
Tissue Inflammation Score
H
Eosinophils/μm

Suppl Fig 3

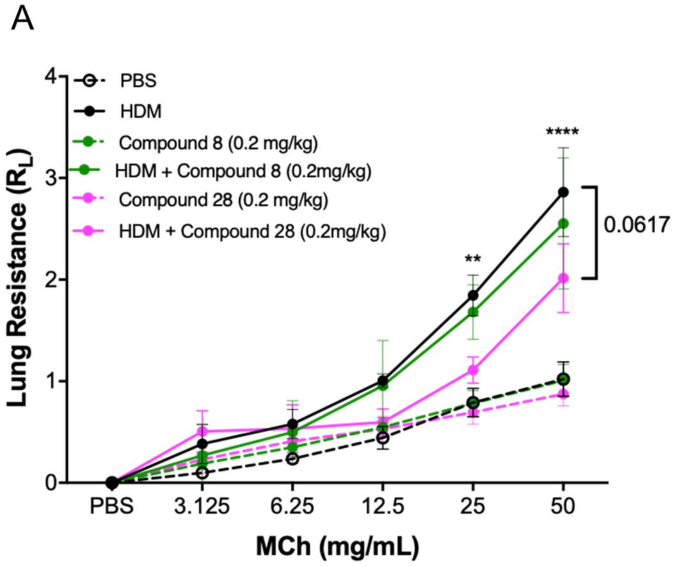

A
Lung Resistance (R_L)
MCh (mg/mL)
PBS
HDM
Compound 8 (0.2 mg/kg)
HDM + Compound 8 (0.2mg/kg)
Compound 28 (0.2 mg/kg)
HDM + Compound 28 (0.2mg/kg)
0.0617
**
****
PBS
3.125
6.25
12.5
25
50

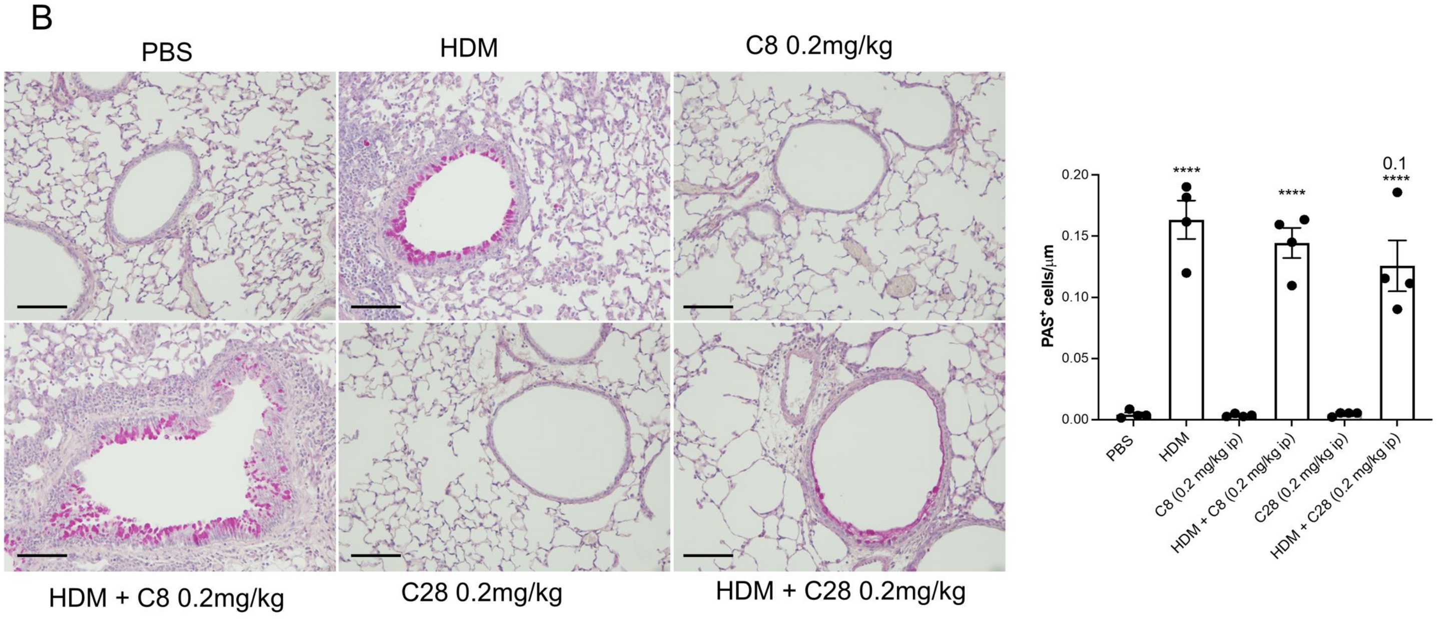

B
PBS
HDM
C8 0.2mg/kg
HDM + C8 0.2mg/kg
C28 0.2mg/kg
HDM + C28 0.2mg/kg
PAS+ cells/μm
0.1
****
PBS
HDM
C8 (0.2 mg/kg ip)
HDM + C8 (0.2 mg/kg ip)
C28 (0.2 mg/kg ip)
HDM + C28 (0.2 mg/kg ip)

Suppl Fig 4

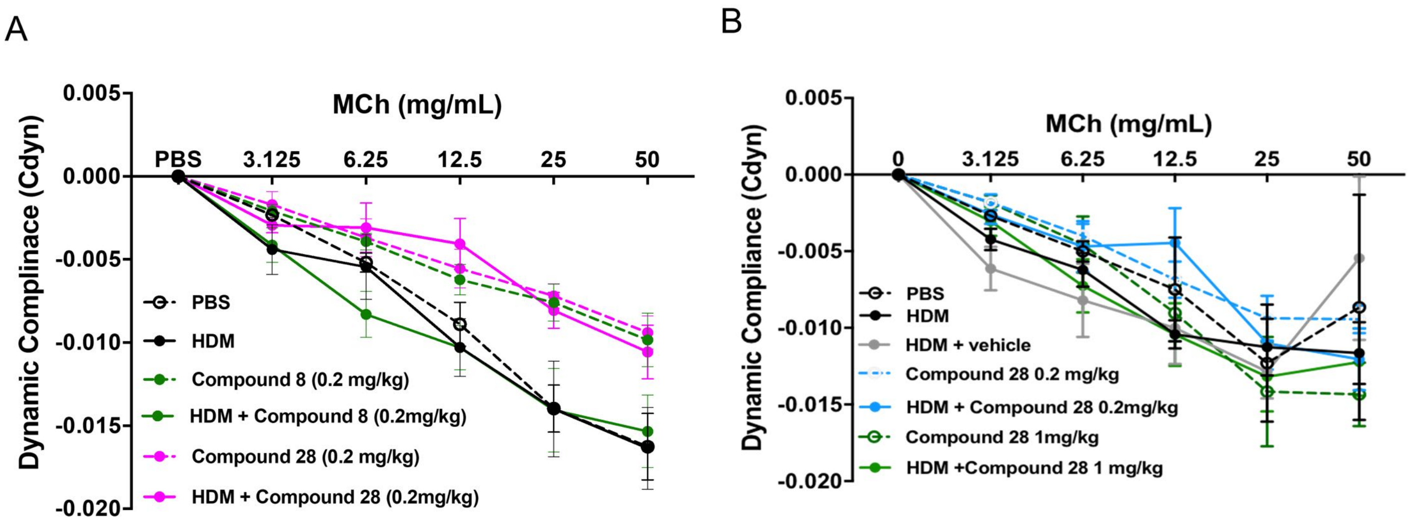
A
MCh (mg/mL)
PBS
3.125
6.25
12.5
25
50
Dynamic Complinace (Cdyn)
0.005
0.000
-0.005
-0.010
-0.015
-0.020
PBS
HDM
Compound 8 (0.2 mg/kg)
HDM + Compound 8 (0.2mg/kg)
Compound 28 (0.2 mg/kg)
HDM + Compound 28 (0.2mg/kg)
B
MCh (mg/mL)
0
3.125
6.25
12.5
25
50
Dynamic Compliance (Cdyn)
0.005
0.000
-0.005
-0.010
-0.015
-0.020
PBS
HDM
HDM + vehicle
Compound 28 0.2 mg/kg
HDM + Compound 28 0.2mg/kg
Compound 28 1mg/kg
HDM +Compound 28 1 mg/kg

Suppl Fig 5

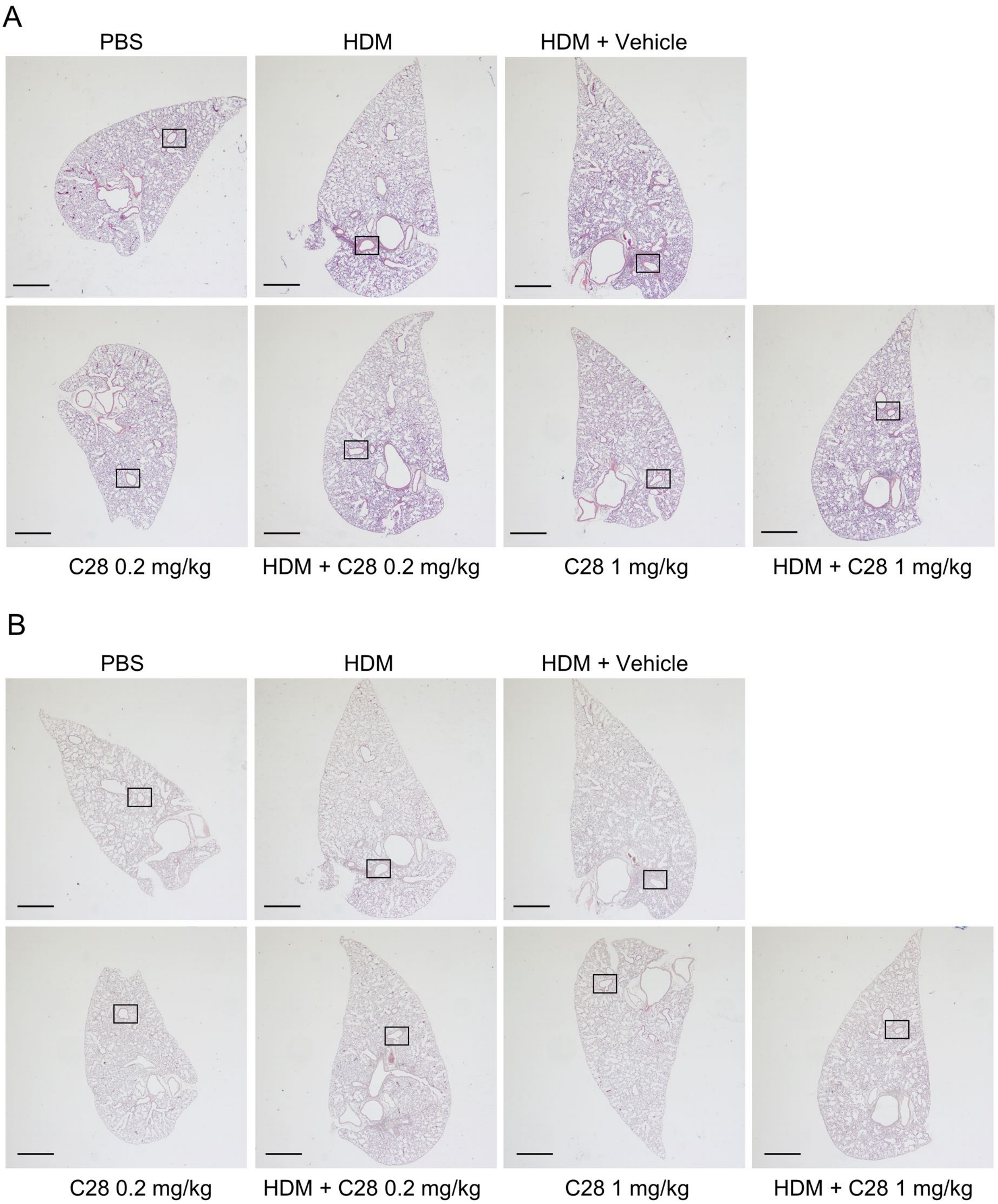
A
PBS
HDM
HDM + Vehicle
C28 0.2 mg/kg
HDM + C28 0.2 mg/kg
C28 1 mg/kg
HDM + C28 1 mg/kg
B
PBS
HDM
HDM + Vehicle
C28 0.2 mg/kg
HDM + C28 0.2 mg/kg
C28 1 mg/kg
HDM + C28 1 mg/kg

Suppl Fig 6

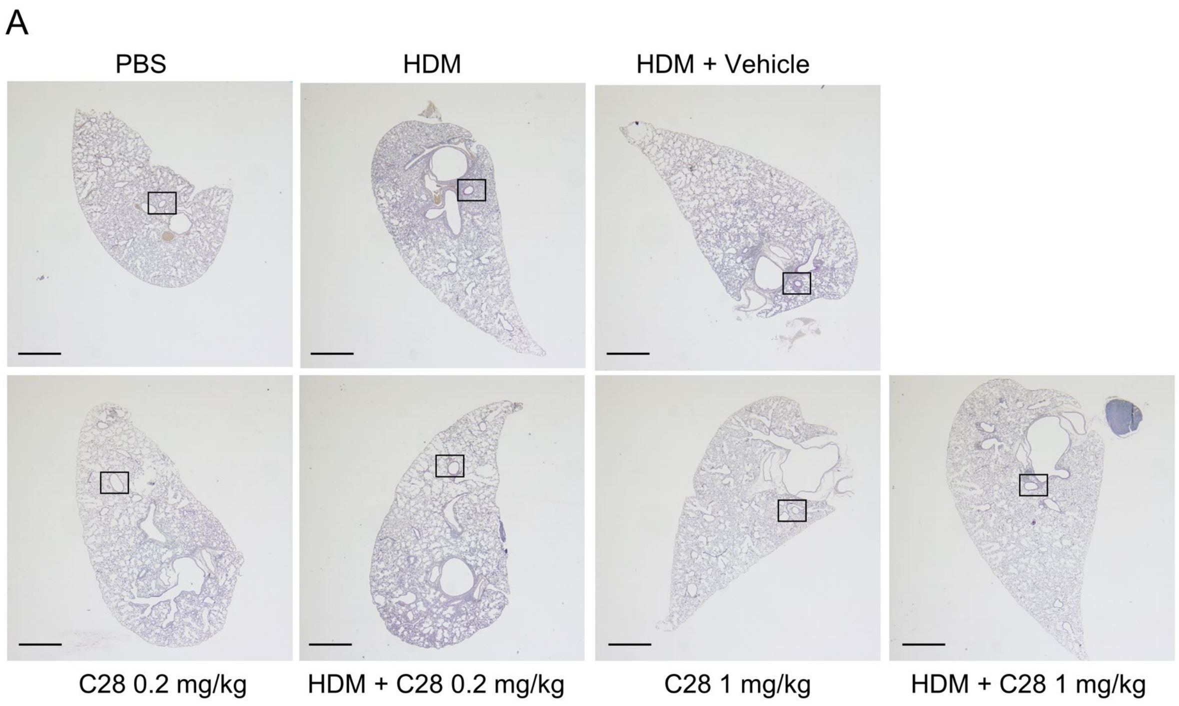
A
PBS
HDM
HDM + Vehicle
C28 0.2 mg/kg
HDM + C28 0.2 mg/kg
C28 1 mg/kg
HDM + C28 1 mg/kg

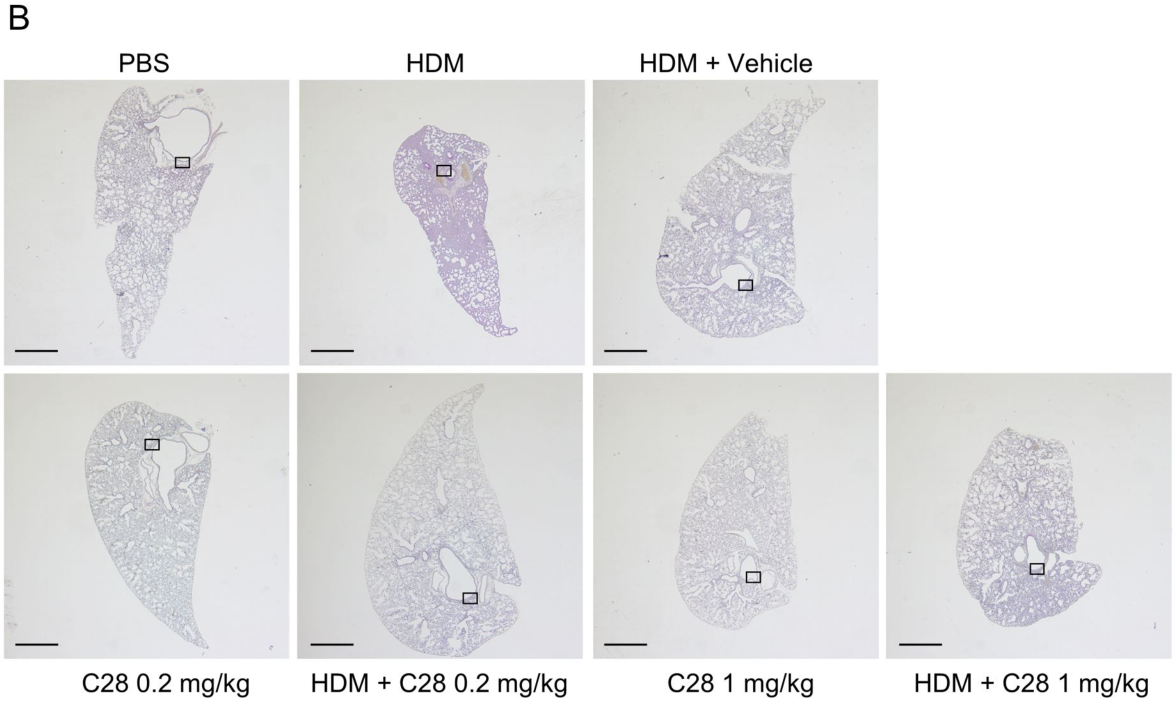
B
PBS
HDM
HDM + Vehicle
C28 0.2 mg/kg
HDM + C28 0.2 mg/kg
C28 1 mg/kg
HDM + C28 1 mg/kg

Supplementary Figure 1: Chemical structures of compound 8(C8) and compound 28 (C28)

Supplementary Figure 2. C28 but not C8 inhibits airway and tissue inflammation in HDM-induced experimental asthma. (A) Mice received either PBS or HDM extract twice a week for 3 weeks. Mice were treated with C8 (0.2 mg/kg) or with C28 (0.2 mg/kg) intraperitoneally 30 min prior to each HDM instillation. Control mice were treated with PBS only. The number of total cells (B), eosinophils (C), lymphocytes (D), neutrophils (E) and macrophages (F) in the bronchoalveolar lavage fluid were quantified. (G) Tissue inflammation was assessed after staining of lung sections with hematoxylin and eosin (H&E); quantification of tissue inflammation by scoring is shown to the right. (H) Tissue eosinophilia was assessed after staining of lung sections with chromotrope 2R; quantification of eosinophils is shown to the right. Data represent mean values ± SEM. *$P \leq 0.05$, **$P \leq 0.01$ and ****$P \leq 0.0001$ vs. the PBS group. #$P \leq 0.05$ and ##$P \leq 0.01$ vs. the HDM group. N = 4 mice per group. HDM = house dust mite. Scale bars: 100 µm.

Supplementary Figure 3. C28 but not C8 partially diminishes AHR and goblet hyperplasia in HDM-induced experimental asthma. Mice received either PBS or HDM extract twice a week for 3 weeks. Mice were treated with C8 (0.2 mg/kg) or C28 (0.2 mg/kg) intraperitoneally 30 min prior to each HDM instillation. Control mice were treated with PBS only. (A) Lung resistance (R_L) was measured using a Buxco FinePointe series instrument. (B) Goblet hyperplasia around the airways in the lung sections was assessed by PAS staining; quantification of eosinophils is shown to the right. Data represent mean values ± SEM. *$P \leq 0.05$, ***$P \leq 0.001$ and ****$P \leq 0.0001$ vs. the PBS group. N = 4 mice per group. HDM = house dust mite. Scale bars: 100 µm.

Supplementary Figure 4. No difference in dynamic compliance (Cdyn) between PBS vs HDM was observed in HDM-induced experimental asthma. Mice received either PBS or HDM extract twice a week for 3 weeks. (A) Mice were treated with vehicle, C8 (0.2 mg/kg) or C28 (0.2 mg/kg) intraperitoneally 30 min before each HDM instillation. Control mice were treated with PBS only. Dynamic compliance (Cdyn) was measured using a Buxco FinePointe series instrument.
(B) Mice were treated with vehicle, C28 (0.2 mg/kg) or C28 (1 mg/kg) intraperitoneally 30 min before each HDM instillation. Control mice were treated with PBS only. Dynamic compliance (Cdyn) was measured using a Buxco FinePointe series instrument.

Supplementary Figure 5. Histological images of lung lobes. Tissue sections of lungs were stained with haematoxylin and eosin (A) or chromotrope 2R (B). Squares indicate areas shown in higher magnification in Figure 2 (A) and Figure 3 (B). Scale bars: 1 mm.

Supplementary Figure 6. Histological images of lung lobes. Tissue sections of lungs were stained with periodic acid-Schiff (PAS) staining. Squares indicate areas shown in higher magnification in Figure 4 (A) and Figure 5 (B). Scale bars: 1 mm.
